# Exploring antioxidant strategies in the pathogenesis of ALS

**DOI:** 10.1515/biol-2022-0842

**Published:** 2024-03-28

**Authors:** Víctor Pinilla-González, Benjamin Montecinos-Barrientos, Clemente Martin-Kommer, Silvia Chichiarelli, Luciano Saso, Ramón Rodrigo

**Affiliations:** Faculty of Medicine, Institute of Biomedical Sciences, University of Chile, Santiago 8380000, Chile; Department of Biochemical Sciences “A. Rossi-Fanelli”, Sapienza University of Rome, 00185 Rome, Italy; Department of Physiology and Pharmacology “Vittorio Erspamer”, Faculty of Pharmacy and Medicine, Sapienza University, P.le Aldo Moro 5, 00185 Rome, Italy

**Keywords:** antioxidants, amyotrophic lateral sclerosis, oxidative stress, iron chelation, multitarget therapy

## Abstract

The central nervous system is essential for maintaining homeostasis and controlling the body’s physiological functions. However, its biochemical characteristics make it highly vulnerable to oxidative damage, which is a common factor in neurodegenerative diseases like amyotrophic lateral sclerosis (ALS). ALS is a leading cause of motor neuron disease, characterized by a rapidly progressing and incurable condition. ALS often results in death from respiratory failure within 3–5 years from the onset of the first symptoms, underscoring the urgent need to address this medical challenge. The aim of this study is to present available data supporting the role of oxidative stress in the mechanisms underlying ALS and to discuss potential antioxidant therapies currently in development. These therapies aim to improve the quality of life and life expectancy for patients affected by this devastating disease.

## 1 Introduction

Amyotrophic lateral sclerosis (ALS) is one of the main causes of motor neuron diseases. These are a group of diseases characterized by musculoskeletal atrophy and sclerosis of the motor pathways of the spinal cord. It corresponds to a neurodegenerative disorder like other diseases such as Alzheimer’s disease, Parkinson’s disease, and other conditions where neurodegeneration is the common marker. ALS, like other neurodegenerative diseases, has no cure, so that when the first symptoms appear, it usually progresses relatively quickly, leading to death from respiratory failure, usually within 3–5 years [1]. In this context, the study of potential therapies to treat or attenuate the progression of the disease has become a longed-for objective for a large part of the scientific population.

### 1.1 Epidemiology

The main epidemiological studies aimed at quantifying the incidence and prevalence of ALS have been carried out on the European continent. These studies have shown that the overall incidence of the disease in Europe is approximately 2–3 persons per year per 100,000 inhabitants [2], slightly higher than studies carried out in the United States, where the annual incidence varies between 1.8 and 2.2 persons per 100,000 inhabitants, according to some studies [3]. As for the differences in incidence by age and gender, it appears to be a disease that affects men and older people more frequently. The overall incidence by gender is 1.3 men for every woman, with the main age group affected being between 65 and 85 years, peaking in the group between 75 and 79 years [4,5].

The established risk factors for the development of the disease are currently accepted to be mainly older age, male sex, and a family history of ALS. However, during the last few decades, the environmental contribution has gained importance, as several studies in Japan during the 1960s and 1970s identified high-incidence clusters located in Guam and the Kii Peninsula [6,7,8]. Recognizing environmental risk factors is extremely important, as they are the only ones that can be modified. However, studies in this area are complex because they require a large amount of funding to quantify the exposures that accumulate over years before the development of the disease. For example, the neurotoxic protein naturally produced by cyanobacteria in some lakes called β-methylamino-L-alanine has been related to increased rates of ALS and Parkinson’s disease [9,10]. Alongside this, exposure to heavy metals and pesticides has also been reported to be associated with an increased risk of developing the disease [11,12,13].

### 1.2 Clinical presentation

The clinical presentation of ALS is characterized by a slow, progressive muscle weakness accompanied by muscle cramps, muscle atrophy, and muscular stiffness. The muscle weakness spreads within the motor system, involving the spinal cord segments and the motor cortex.

The typical presentation of the disease features unilateral distal weakness and muscular atrophy in the upper and lower limbs or bulbar muscles. In the upper limb, it usually affects the dominant hand’s thenar muscles, while in the lower limb, it affects the anterior tibial muscle. When the bulbar muscles are affected, the patient may experience dysarthria or dysphagia. In the later stages of the disease, it may present with a head drop or postural problems [14]. Some of the symptoms that may appear during the course of the disease include muscular weakness, sialorrhea, bronchial secretions, pseudobulbar condition, cramps, and spasticity [15].

There is no specific test suitable for diagnosing the disease. Therefore, it is based on clinical symptoms, physical examination, and electromyography. It must also meet certain criteria of the World Federation of Neurology [14]. To diagnose ALS, there must be the presence of positive criteria of lower motor neuron (LMN) signs, upper motor neuron signs, progression of symptoms and signs, and the absence of sensory signs, sphincter disturbances, visual disturbance, autonomic features, basal ganglion dysfunction, Alzheimer-type dementia, and ALS mimic syndromes. Additionally, the diagnosis is supported by fasciculations in one or more regions, neurogenic changes in electromyography results, normal motor and sensory nerve conduction, and the absence of conduction block.

There are recommended mandatory tests such as blood tests, electromyography, nerve conduction velocity, cranial/cervical, thoracic, and lumbar magnetic resonance, and finally, a chest X-ray. A definite ALS diagnosis is based on the following criteria: LMN and UMN clinical signs or electrophysiological evidence in three regions, UMN and/or LMN clinical signs in one region, and the patient is a carrier of a pathogenic SOD1-gene mutation. A clinically probable ALS is based on UMN and LMN clinical or electrophysiological evidence by UMN and LMN signs in two regions, with some UMN signs rostral to the LMN signs. Finally, clinically possible ALS is based on UMN and LMN clinical or electrophysiological signs in one region only, or UMN signs in at least two regions, or UMN and LMN signs in two regions with no rostral UMN signs rostral to LMN signs, or neuroimaging and laboratory studies have excluded other diagnoses [16].

## 2 Oxidative stress and pathophysiology of lateral amyotrophic sclerosis

### 2.1 Oxidative stress and antioxidant defense system

Oxidative stress is a prevalent mechanism found in several pathological conditions, characterized by an imbalance between the production of reactive oxygen species (ROS) and/or reactive nitrogen species and the antioxidant capacity of the cell. This imbalance disrupts redox homeostasis and causes damage at the molecular level [17]. The most important free radicals include oxygen-derived species such as the superoxide anion (O_2_^−^), hydrogen peroxide (H_2_O_2_), and the hydroxyl radical (OH^−^), as well as nitrogen-derived species such as nitric oxide (NO^−^), nitrogen dioxide radical (NOO^−^), and peroxynitrite anion (ONOO^−^) [18]. These molecules play essential roles in processes such as microbial defense, activation of transcription factors, and protein phosphorylation at low concentrations. However, in excess, they become harmful because they possess unpaired electrons in their outer orbitals that react with other molecules, altering their structures [19,20].

Cells generate free radicals enzymatically and spontaneously from various sources. These sources include oxidative phosphorylation in the inner mitochondrial membrane (OXPHOS), NADPH oxidase (NOX) in activated leukocytes during the respiratory burst process, as well as myeloperoxidase and uncoupled nitric oxide synthase (uncNOS) [21,22]. The most prominent non-enzymatic pathways involve transition metal ions, such as iron or copper, which contribute to the production of free radicals. Notably, the Fenton and Haber-Weiss reactions are examples of reactions in which ROS are generated from free ionic iron [23].

Given the damage caused by ROS, cells have developed various defense mechanisms to counteract their effects, classified as enzymatic and non-enzymatic antioxidants. Enzymatic antioxidants comprise superoxide dismutase (SOD), catalase (CAT), glutathione peroxidase (GPX), and thioredoxin [24,25,26,27,28,29]. Their expression is mainly regulated by Nuclear factor erythroid-2-related factor 2 (Nrf2), typically inhibited by the adaptor protein Keap1 under normal conditions. However, oxidative stress induces a conformational change in Keap1, releasing Nrf2. Consequently, Nrf2 translocates into the cell nucleus, binding to promoter regions called antioxidant response elements, leading to the transcription of genes responsible for detoxification, antioxidant protection, protein turnover, and other functions [30,31,32].

Non-enzymatic antioxidants can be divided into endogenously produced substances, such as glutathione (GSH), uric acid, and bilirubin, and those introduced externally through diet or supplements, such as water-soluble vitamin C or fat-soluble vitamins A and E, among others [33].

### 2.2 Pathophysiology of ALS

**Figure 1:**
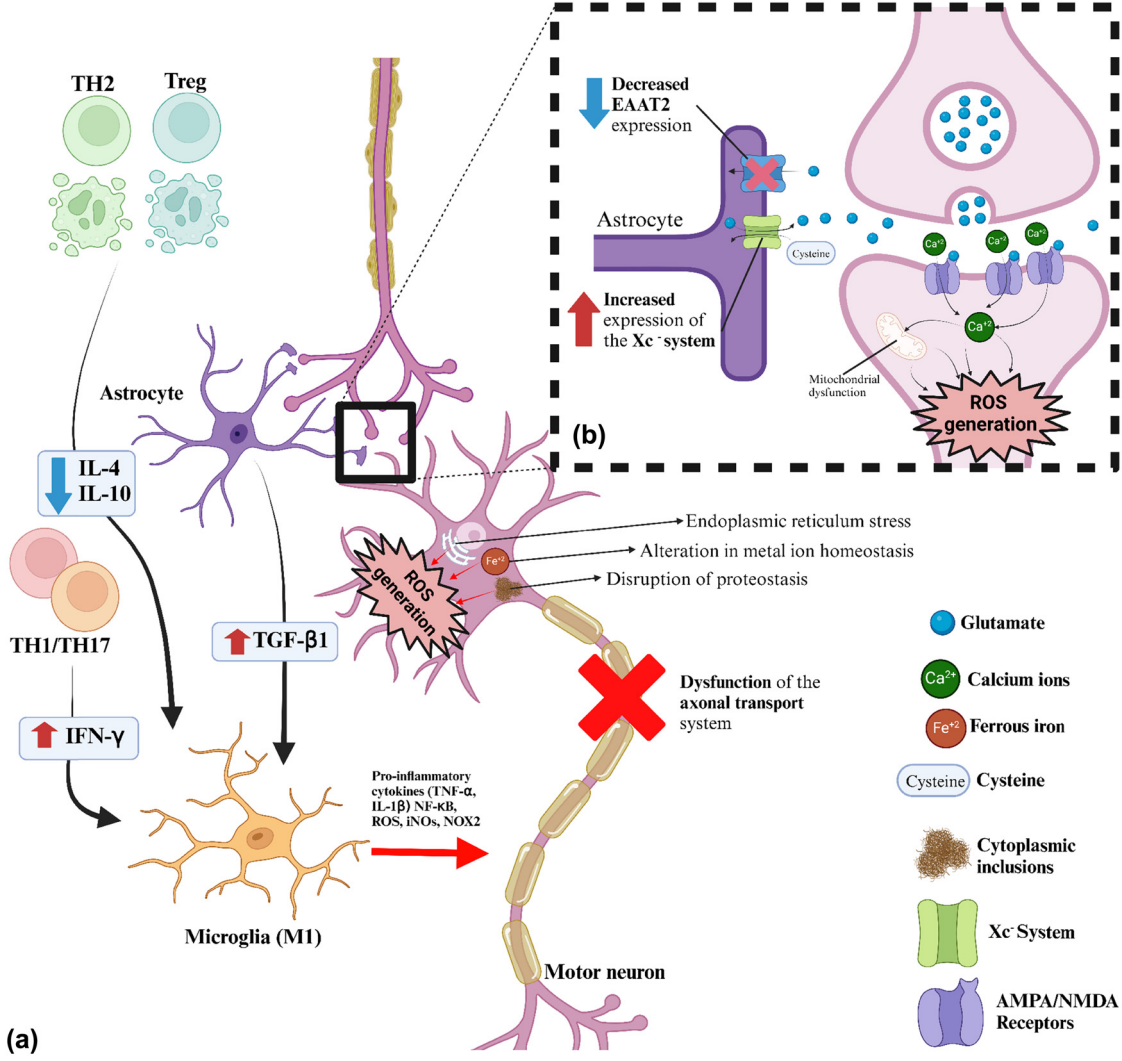
Summary of the main pathogenic pathways involved in ALS. (a) Schematic diagram showing the neurotoxic effects of neuroinflammation in ALS and the main alterations in the cytoplasm, such as reticulocyte stress, altered metal ion homeostasis, disruption of proteostasis, and axonal transport dysfunction. The main events that characterize neuroinflammation in ALS include decreased activity of TH2-like cells and regulatory T lymphocytes (Treg), as well as increased migration of proinflammatory T cells (TH1/TH17). These cells interact with microglia (M1 subtype) and astrocytes, leading to motor neuron damage. (b) Enlargement of a synapse illustrating the mechanism of excitotoxicity due to excessive glutamatergic activity and the involvement of astrocytes in this process. Red upward arrows (↑) or blue downward arrows (↓) indicate upregulation or downregulation. Transforming growth factor beta 1 (TGF-β1), tumor necrosis factor alpha (TNF-α), ferrous iron (Fe^2+^), sodium ions (Na^+^), calcium ions (Ca^2+^), interleukin 1 beta (IL-1β), interleukin 4 (IL-4), T helper lymphocytes (TH1, TH2, TH17), regulatory T lymphocyte (Treg), inducible nitric oxide synthase (iNOS), NADPH oxidase 2 (NOX 2), hydrogen protons (H^+^), oxygen free radicals (ROS), alpha-amino-3-hydroxy-5-methyl-4-isoxazolepropionic acid (AMPA) receptor, N-methyl-D-aspartate (NMDA) receptor, excitatory amino acid transporter 2 (EAAT2), and cysteine/glutamate antiporter system (Xc^−^ System).

ALS is characterized by the progressive degeneration of the pyramidal motor neurons in the corticospinal pathway located in the motor cortex, brainstem, and spinal cord. The etiology is typically sporadic and of unknown cause, with only a small percentage attributed to inherited genetic abnormalities in specific genes. The affected biochemical pathways mainly involve altered metal ion homeostasis, excitotoxicity, protein quality control failure, and neuroinflammation (Figure 1). In this context, oxidative stress serves as a common biomarker for the disruption of these various biochemical pathways, playing a significant role in promoting damage and contributing to the progression of the disease.

#### 2.2.1 Alteration in metal ion homeostasis

Metal ions are widely distributed throughout the brain and play pivotal roles in maintaining normal CNS functions. Among the most important of these metals are transition metals such as iron (Fe), copper (Cu), and zinc (Zn), which exhibit redox activity. For instance, iron is essential for myelin synthesis, participates in oxidative phosphorylation, neurotransmitter production, oxygen transport, and numerous other functions [34]. On the other hand, copper contributes to neuronal excitability and serves as a cofactor for the enzyme cytochrome c oxidase in the mitochondria. Additionally, it is involved in the antioxidant enzyme type 1 superoxide dismutase (SOD1), along with zinc (Zn) [35].

As mentioned, iron plays various crucial roles in the CNS; however, to fulfill these functions, it must traverse the blood–brain barrier (BBB). In this process, the ferric form of iron (Fe^3+^), bound to transferrin, interacts with the membrane protein transferrin receptor 1 (TfR1) on the endothelium of the BBB, inducing endocytosis [36]. Subsequently, Fe^3+^ iron is released from transferrin and reduced to ferrous iron (Fe^2+^) through the action of the enzyme ferrireductase, accompanied by gradual endosome acidification. Following this, Fe^2+^ exits the endothelium via ferroportin and can be taken up by brain transferrin, which is primarily synthesized by oligodendrocytes. Notably, these cells accumulate most of the iron in the CNS [37,38]. Once inside neuronal cells, Fe^2+^ is retained in the labile iron pool and can be re-oxidized to Fe^3+^ by H_2_O_2_ through the Fenton reaction. Excess iron is typically stored in cytosolic ferritin [39]. Therefore, maintaining the redox balance of iron is critical for its uptake, transport, and storage.

Regarding the link between iron and ALS, multiple studies have reported dysregulation of different iron metabolism markers in ALS patients. This includes iron accumulation in the CNS, decreased transferrin levels, and increased serum ferritin levels [40,41,42]. Despite this, it has proven challenging to unify these findings into a single model that explains the pathogenic role of iron in ALS. However, one potential candidate seems to be ferroptosis, a type of programmed cell death initiated by oxidative disruptions in the extracellular microenvironment. It is under the constitutive control of GPx4 and can be inhibited by iron chelators and lipophilic antioxidants [43]. Ferroptosis occurs due to alterations in the intracellular Fe^2+^ homeostasis and a decrease in the cell’s antioxidant defenses, causing ferrous iron to generate free radicals via the Fenton reaction, leading to lipid peroxidation and cell death. Some markers of ferroptosis in ALS patients supporting its involvement in the disease include GPx4 depletion in post-mortem spinal cord tissue in patients with both sporadic and familial ALS, along with increased markers of lipid peroxidation, such as 4-hydroxynonenal in plasma and cerebrospinal fluid (CSF) [44,45].

On the flip side, copper assumes vital roles in the CNS, acting as a cofactor in multiple oxidoreductase enzymes, participating in the electron chain, neurotransmitter synthesis, and myelination, acting as an eliminator, and contributing to iron homeostasis by facilitating the conversion of Fe^2+^ to Fe^3+^ through binding to ceruloplasmin [35,46,47]. In the context of its connection to the pathophysiology of ALS, it is postulated that this is primarily due to mutations in the enzyme SOD1, which relies on copper as a significant cofactor [48]. Examination of the catalytic site in transgenic mouse models with SOD1 mutations has revealed a copper deficiency at this site [49]. In this context, a therapy currently under investigation for ALS is the use of Cu^II^(atsm), demonstrated to facilitate the incorporation of copper into SOD1 [50]. This process transforms the abnormal, copper-deficient SOD1 into its physiologically mature, copper-rich form. Although this drug has shown promising results in animal models, its actual implication in non-SOD1-related ALS cases is still unknown, as this mutation only explains approximately 2% of total cases. Nevertheless, studies on sporadic ALS have been conducted, unveiling copper deficiencies in other cuproenzymes like ceruloplasmin, suggesting that treatment with Cu^II^(atsm) could be a potential therapy [51]. However, further research is essential to evaluate the true role of copper in the disease’s pathogenesis and its potential therapeutic targets.

#### 2.2.2 Excitotoxicity

Glutamate is the most abundant excitatory neurotransmitter in the central nervous system. It is released from presynaptic neurons into the synaptic cleft, leading to the activation of ligand-gated ion channels (ionotropic receptors) and G protein-coupled receptors (metabotropic receptors). This results in depolarization of the presynaptic neuron due to the influx of sodium (Na^+^) and calcium (Ca^2+^) and the outflow of potassium (K^+^). The action of glutamate is terminated by reuptake systems located in the astrocytes that surround the synapses. In astrocytes, glutamate is converted into glutamine, which lacks neurotransmitter properties and can be released and then available to neurons for conversion back to glutamate via a glutamine reuptake system [52].

The most important glutamate receptors are the ionotropic NMDA and AMPA receptors, which are permeable to Na^+^ entry and K^+^ exit. The permeability of these channels to calcium is different; NMDA channels are always permeable to Ca^2+^ influx, while AMPA receptors depend on the presence of the GluR2 subunit, which, if present, makes them impermeable [53]. Under resting conditions, the NMDA channel is blocked by magnesium ions, which is reversed following depolarization generated by the activation of AMPA channels.

In this context, excitotoxicity occurs when there is a prolonged excitatory glutamate response, resulting in a massive influx of calcium into the cell. This increase in intracellular Ca^2+^ concentration can be initially buffered by the mitochondria and endoplasmic reticulum (ER). However, these systems can become overwhelmed. Increased Ca^2+^ in the mitochondria leads to the depolarization of their membrane, disrupting the respiratory chain. Additionally, overactivation of ATP-dependent ion pumps in an attempt to restore ionic homeostasis results in ATP depletion. Energy stress is enhanced by the activation of catalytic enzymes and the generation of free radicals that cause further cellular damage. For example, Ca^2+^ increases the activity of NOX, uncNOS, as well as calpains that cleave cytoskeletal proteins, transporters, and membrane receptors [54,55]. All of this ultimately leads to neuronal necrosis.

In this regard, several studies have attempted to elucidate the mechanism of ALS excitotoxicity. Motor neurons have been shown to be remarkably sensitive to this process and vulnerable to oxidative stress derived damage. This sensitivity arises from their large size, approximately 100 μm in diameter, and axons reaching up to 1 m in length, involving high oxygen and energy demands and increased ROS production. Unfortunately, this alteration is not currently accompanied by enough increased activity of antioxidant enzymes to recover a redox balance. Additionally, motor neurons express high levels of Ca^2+^-permeable AMPA receptors and low levels of Ca^2+^ transporter proteins that otherwise could help buffering the increase in cytoplasmic Ca^2+^ concentration [56].

Regarding the origin of alterations in glutamate signaling in ALS, astrocytes have been proposed as one of the main cells involved in the process. For example, some studies reported a decrease in the EAAT2, which is involved in glutamate reuptake in astrocytes [57,58]. In addition, there is an increase in the expression of the cysteine/glutamate antiporter system (Xc^−^ system), which primarily serves to support the antioxidant response by regenerating reduced GSH. While this increase may be a compensatory mechanism for the oxidative stress triggered by the disease, it also causes an enhanced glutamate, thereby increasing excitotoxicity [57]. This molecular pathway interacts with transition metal imbalances. For example, it has been observed that iron overload in astrocytes induces cytoplasmic Ca^2+^ imbalance toward increased concentration, which could also impair neuronal function [59].

Another factor that has been studied in relation to ALS is the degeneration of the serotonergic system, which could facilitate glutamate toxicity. This is because serotonin, in physiological conditions, acts as a facilitator of neuronal depolarization by glutamate. It has also been observed that serotonin stimulates the expression of the GluR2 subunit of AMPA, which would increase calcium impermeability [60,61,62,63]. Nevertheless, there is still a lack of information to fully understand the relationship between the serotonergic system and the pathogenesis of ALS.

#### 2.2.3 Disruption of proteostasis

The proteome is typically understood as the set of proteins expressed in a given cell during its lifespan. Proteostasis, or protein homeostasis, refers to the maintenance of each protein in the specific proteome of each cell type, ensuring it remains in the necessary conformation, concentration, and location for proper cellular function. The proteostasis network encompasses pathways that regulate protein biogenesis, trafficking, and degradation [64,65]. In ALS, this network is significantly impaired, with various steps in this process failing, leading to the loss of protein functions and the pathological accumulation of proteins in the neuronal cytoplasm. For instance, the DNA-binding protein TAR (TDP-43) is one of the main cytoplasmic inclusions found in both familial and sporadic ALS. In the latter case, it may be linked to enterovirus infection [66,67]. Another significant constituent of the cytoplasmic inclusions in ALS includes SOD1, Fused in Sarcoma, among many others [68,69]. The loss of these functions ultimately leads to neuronal death.

The formation of pathological protein aggregates, which are part of the intermediate filaments of the cytoskeleton, is a common pathological feature in ALS [70]. The primary function of these proteins is to participate in axonal transport. In ALS, these proteins are hyperphosphorylated, and their dynamic assembly is impaired. This pathophysiological feature predominantly affects motor neurons, given that, as mentioned earlier, they have axons that can extend up to 1 m in length, making axonal transport vital [71].

Another crucial element triggered by the accumulation of pathological aggregates is ER stress. This phenomenon is characterized by the ER’s attempt to repair misfolded proteins by reducing protein synthesis, increasing chaperone expression, and activating the ER-associated degradation pathway. Unfortunately, this response is insufficient to compensate for the increased misfolded proteins, leading to the generation of free radicals and the release of Ca^2+^ into the cytosol, thereby enhancing the above-mentioned damage pathways [72,73].

#### 2.2.4 Neuroinflammation

Neuroinflammation refers to the responses of glial cells, such as astrocytes and microglia, as well as cells of the circulating immune system, such as monocytes, neutrophils, and lymphocytes, when they interact with nerve cells in the central nervous system during situations of infection, injury, or degeneration. Initially, this acute response may be beneficial; however, if these cells fail to repair the damage, they maintain their reactive state and continue to recruit astrocytes and microglia, resulting in an ongoing inflammatory process that leads to damage progression [74].

In this context, post-mortem anatomopathological studies and positron emission tomography imaging of ALS patients have shown evidence of glial cell proliferation and activation, as well as T-cell infiltration in areas affected by ALS, indicating the pathogenic role of each of these cells in the progression of ALS [75,76].

One of the cells involved is microglia, which is a resident macrophage in the CNS and has the capacity to exist in various states, depending on its interaction with the environment. It is typically in an inactive state; however, certain signals can lead to its activation and the acquisition of an inflammatory (M1) or anti-inflammatory (M2) phenotype. The M2 phenotype is characterized as protective, producing anti-inflammatory cytokines and neurotrophic factors, while the M1 phenotype is toxic and capable of inducing ROS generation, as well as proinflammatory cytokines [77]. Concerning its involvement in ALS, it has been observed that in pre-symptomatic states, microglia are found in the M2 state, overexpressing neurotrophic factors and anti-inflammatory cytokines such as interleukin-10 (IL-10). As the disease progresses, it acquires an M1 phenotype with increased activity of pro-oxidant enzymes such as NOX, inducible nitric oxide synthase (iNOs), and the release of cytokines such as tumor necrosis factor alpha (TNF-α), and IL-1β [78]. Regarding stimuli conditioning the microglia phenotype, IL-4 has been shown to have the ability to divert microglia toward an M2 phenotype, improving clinical outcomes in the early stages of ALS [79].

Astrocytes are other cell types involved, being the most abundant glial cells in the CNS. Their main role is to provide nutrients and support to neurons, as well as to maintain the impermeability of the BBB. However, they also have the capacity to regulate the immune system by secreting cytokines, communicating with neighboring microglia, and infiltrating immune cells [80]. Regarding their involvement in ALS, it has been observed that these cells acquire a neurotoxic phenotype characterized by direct and indirect damage to motor neurons. Direct damage occurs by secreting toxic soluble factors such as inflammatory cytokines and ROS, as well as by interacting with altered motor neuron receptors, such as type 1 major histocompatibility complex [81,82]. On the other hand, indirect damage is exerted by interacting with microglia and T cells. For example, overexpression of transforming growth factor beta (TGF-β1) by astrocytes interferes with the neuroprotective response of microglia, as does the expression of nuclear factor κB (NF-κB) in astrocytes during the symptomatic phase, which promotes microglial proliferation and accelerates disease progression [83,84].

Finally, another important cellular component that modulates the inflammatory response at the CNS level in ALS is T lymphocytes. These cells have basically two subpopulations: CD4+ and CD8+. In ALS, both subpopulations are actively involved. For example, CD4+ T cells in early stages acquire an anti-inflammatory TH2 and Treg phenotype, which, through the secretion of IL-4, induces an M2 phenotype in microglia. In advanced stages, they acquire a TH1/TH17-type inflammatory phenotype, releasing inflammatory cytokines such as interferon gamma, ROS, and NO, enhancing the neurotoxic effects of M1 microglia [85]. In ALS patients, peripheral blood analysis shows that Treg and Foxp3 levels correlate negatively with disease progression [86]. In addition, CD8+ T cells also appear to have a dual role in neuroprotection and cytotoxicity. For example, their infiltration of the peripheral nervous system has been associated with myelin regeneration along the motor axon and at the neuromuscular junction, thereby delaying muscle denervation and prolonging survival. Their infiltration in the spinal cord, however, is neurotoxic to motor neurons [87,88].

In summary, various components of the immune system interact with each other at different stages of the disease, modulating neuroinflammation in ALS. A detailed understanding of the role of each cell remains an open question, but once clarified, it could open the door to the development of therapies for the treatment of ALS.

### 2.3 Potential antioxidant therapies

#### 2.3.1 Actual therapy

There is currently no cure for ALS; however, there are two FDA-approved treatments that aim to alleviate symptoms: riluzole and edaravone. The former is an anti-glutamatergic drug that blocks postsynaptic glutamate receptors, reduces glutamate release, and inactivates voltage-dependent sodium channels [89]. Riluzole has been shown to prolong patients’ lives by 3–6 months. Riluzole acts as a glutamate antagonist and significantly influences the survival rate of ALS, slowing the course of the disease progression. Its mechanism of action includes inhibition of presynaptic glutamate release, increased clearance, glutamate receptor antagonism, normalization of sodium channel function, reduction of cortical hyperexcitability, and stimulation of growth factor synthesis to promote neuronal branching [90,91].

In turn, edaravone is a free radical scavenging antioxidant that targets peroxyl radicals by scavenging free radicals, thereby reducing oxidative stress and protecting motor neurons from oxidative damage. Edaravone inhibits the formation of linoleic acid hydroperoxide via hydroxyl radicals generated by the Fenton reaction rather than hydroperoxide itself. It has also been reported that edaravone does not react with superoxide anion radicals and is lipophilic, so it is able to diffuse passively across membranes to scavenge lipid peroxide radicals [92,93]. These treatments offer important options for ALS patients.

#### 2.3.2 Opportunities for antioxidant treatment

It has been suggested that antioxidants could serve as a potential therapy for ALS, given their ability to alleviate oxidative stress, a factor implicated in the disease’s pathogenesis. Numerous studies have delved into the therapeutic potential of various antioxidant compounds in ALS, such as vitamin E, *N*-acetyl-l-cysteine (NAC), coenzyme Q10 (CoQ10), flavonoids, iron chelators, among others. However, despite encouraging results in preclinical studies, the transition from antioxidant therapies to clinical application has proven challenging.

Many antioxidant compounds that demonstrated promising results in preclinical research have failed to showcase therapeutic benefits in clinical trials involving ALS patients. Against this backdrop, the subsequent section will detail the primary antioxidants used, along with a brief explanation of their mechanism of action and the key clinical and preclinical findings.

##### 2.3.2.1 Vitamin E

Vitamin E, a fat-soluble vitamin, exists in eight different forms (α-, β-, γ-, δ-tocopherol, and α-, β-, γ-, δ-tocotrienol), with δ-tocopherol being the most commonly utilized form. Being fat-soluble, it is predominantly concentrated in cell membranes, where it performs its antioxidant role by restricting lipid peroxidation of polyunsaturated fatty acids [94]. In addition to its antioxidant action, an anti-inflammatory effect has been observed, mainly through the inhibition of the enzyme cyclooxygenase-2 (COX-2), which limits the formation of prostaglandin E2 [95]. These properties have prompted research into the possible role of vitamin E supplements in the treatment of ALS.

In this context, preclinical studies with murine models of various neurodegenerative diseases have demonstrated that vitamin E plays both a neuroprotective and a neuroregenerative role [96]. In addition to this evidence, an *in vitro* study with human-induced pluripotent stem cell-derived motor neurons (hiMN) showed the potential of vitamin E to attenuate ferroptosis in motor neurons, a process closely related to the pathogenesis of the disease [97].

Regarding clinical studies in ALS patients, there are currently not a large number of them, and those that have been conducted often have contradictory results, making it difficult to draw solid conclusions. For example, some observational studies have identified an inverse relationship between vitamin E levels and disease development and progression, but these results have not been fully replicated [98,99,100,101,102]. Concerning clinical trials in ALS patients, their number is limited, and the results are not very encouraging. For example, a study that attempted to investigate the efficacy of treatment with high-dose vitamin E as an adjunct to riluzole in ALS patients failed to obtain satisfactory results, as no significant differences were found compared to the control group [103]. Thus, before adopting the use of vitamin E as a viable therapeutic option, further human studies should be conducted to determine its role in the course of the disease, as well as to determine the effective dose and safety of its use.

##### 2.3.2.2 NAC

NAC is an acetylated derivative of the amino acid cysteine, widely employed in clinical and experimental contexts as an antioxidant. Its capability to act as a precursor in the synthesis of GSH makes it particularly valuable in replenishing GSH consumed by free radicals [104,105]. This attribute, coupled with its indirect role as a metal ion chelator and its anti-inflammatory potential through NF-кB inhibition, positions it as a potential candidate for treating ALS [106,107]. Preclinical *in vitro* and *in vivo* studies have demonstrated that NAC suppresses oxidative stress and inflammation, showcasing benefits in survival and motor performance in murine models of ALS [108,109].

In the realm of human clinical trials, the availability of results is limited and, unfortunately, not very encouraging. These studies have failed to demonstrate that NAC administration in ALS patients is associated with significant changes in survival or disease prognosis [110,111]. Difficulty in achieving adequate concentrations in the brain and spinal cord due to the BBB has been proposed as a possible explanation for these unfavorable outcomes. Given this limitation, some researchers have explored the intranasal route as a non-invasive option, with promising results in animal studies showing an increase in survival in mouse models of ALS, while testing in humans is still pending [112].

In light of these results, while NAC has the potential to play a protective role in ALS, more human studies are needed to determine the effective dose as well as the optimal route of administration.

##### 2.3.2.3 CoQ10

CoQ10, also known as ubiquinone, is a lipophilic molecule primarily located in the electron transport chain within the inner membrane of mitochondria. However, its function extends beyond this, as it serves as a potent antioxidant safeguarding cell membranes, thus limiting lipid peroxidation and ferroptosis [113,114]. In the context of ALS, an elevation in plasma oxidized CoQ10 concentration has been noted in ALS patients, suggesting that supplementation might offer therapeutic benefits [115]. Preclinical studies, particularly in murine models of the disease, have demonstrated increased survival with CoQ10 supplementation [116].

These findings have spurred clinical studies in humans to evaluate the efficacy and tolerability of CoQ10 supplementation. While the administration of large doses is generally well-tolerated, clinical effectiveness remains uncertain due to conflicting results [117]. For instance, a phase II trial assessing daily CoQ10 supplementation in ALS patients found no significant difference between the CoQ10 group and the placebo group, indicating insufficient evidence to support a phase III trial [118]. The inconsistency in results has hindered the utilization of CoQ10 as a therapy in ALS, emphasizing the necessity for additional studies to determine the role of CoQ10 in the pathogenesis of ALS.

##### 2.3.2.4 Polyphenols

Polyphenols are secondary metabolites primarily produced by plants, and as such, they are present in various fruits, flowers, leaves, and tree bark [119]. Renowned for their antioxidant and anti-inflammatory properties, polyphenols have found applications in various diseases, including ALS, as evidenced by the study of certain polyphenols [120]:

###### 2.3.2.4.1 Quercetin

Quercetin, a polyphenol within the flavonoid group, is found in abundance in fruits such as grapes and vegetables such as onions [121]. This substance exhibits remarkable neuroprotective properties attributable to its antioxidant, anti-apoptotic, and anti-inflammatory properties [122]. In addition, it is actively involved in enhancing mitochondrial function and exhibits iron-chelating abilities, making it an effective agent against ferroptosis [121].

In this context, various mechanisms have been investigated through *in vitro* and *in vivo* studies to evaluate the potential therapeutic properties of quercetin for ALS. Notably, its inherent capacity to reduce free radicals and its ability to positively modulate silent information regulator 1 (SIRT1), which contributes to decreasing ER stress, apoptosis, inflammation, and limiting neuronal death resulting from excitotoxicity, are significant [123,124,125].

Additionally, quercetin has been found to have the ability to attenuate the formation of toxic SOD1 fibrils, which are implicated in the disease [126,127,128]. Despite these promising results, the application of quercetin in human clinical trials has been limited due to its physicochemical properties, which hinder the achievement of optimal concentrations in the central nervous system [129]. Given this challenge, research on nanoformulations has recently been initiated to improve its bioavailability; however, safety and efficacy studies in the context of ALS have yet to be conducted [130].

###### 2.3.2.4.2 Curcumin

Curcumin is a polyphenol present in the medicinal herb known as turmeric (*Curcuma longa*). This compound exhibits significant potential as a pharmacological agent, playing a pivotal role in addressing oxidative stress and modulating inflammatory responses. Curcumin can positively impact antioxidant defenses by activating the adenosine monophosphate-activated protein kinase (AMPK) pathway, which, in turn, triggers Nrf2 signaling [131]. Regarding the regulation of inflammatory responses, curcumin’s effectiveness lies in its ability to inhibit the NF-κB pathway, a critical element in the inflammatory process [131].

In terms of its potential application as a therapy for ALS, several clinical trials have been conducted. One noteworthy double-blind clinical trial revealed that curcumin supplementation could lead to a slight slowing of disease progression. Additionally, improvements in aerobic metabolism and reductions in oxidative damage were observed in ALS patients [132]. This trial is complemented by another clinical study utilizing a nanotechnology-based formulation to enhance protection against ALS. The results suggest that nanocurcumin increased survival rates in treated patients compared to the control group [133]. Despite these promising findings, larger-scale clinical studies are still necessary to obtain more representative results. It is also crucial to determine the most appropriate doses and routes of administration for curcumin as a therapy in the context of ALS.

###### 2.3.2.4.3 Resveratrol

Resveratrol, a natural polyphenol with antioxidant properties, has shown promising effects in treating ALS. Multiple preclinical studies involving animal models and *in vitro* cell culture assays suggest that resveratrol can delay disease onset, extend lifespan, and improve motor neuron function in ALS by activating SIRT1 through AMPK activation, much like quercetin [134,135]. Furthermore, recent research has found that resveratrol can correct the pathological state of NF-κB acetylation, a factor implicated in the pathogenesis of ALS [136].

Although these findings suggest that resveratrol may be a promising therapeutic intervention for ALS, clinical trials in ALS patients demonstrating the effects described in the literature and evaluating the safety of resveratrol in specific clinical settings are still lacking.

##### 2.3.2.5 Melatonin

Melatonin is a neurohormone synthesized by the pineal gland that has garnered significance due to its outstanding antioxidant capacity. It acts as a direct free radical scavenger and enhances antioxidant defenses by triggering GSH synthesis, along with the activation of antioxidant enzymes like SOD and GPx [137,138]. This capability has prompted the exploration of melatonin as a potential therapy for ALS. Some preclinical studies have indicated that melatonin inhibits cytochrome c release, prevents cell death, and delays disease onset and progression in mouse models of ALS [139]. However, the validation of these findings encounters challenges, as certain studies with murine models have not affirmed its neuroprotective role [140].

In terms of outcomes from clinical trials involving ALS patients, a systematic review analyzing 23 clinical trials employing melatonin discovered that its usage is linked to slower disease progression and prolonged patient survival. Nevertheless, this review faced notable limitations, including the retrospective nature of the analysis, a small sample size, and the subjectivity of the databases employed [141]. In this context, definitive conclusions regarding the application of melatonin in ALS remain elusive, given the persisting controversial results.

##### 2.3.2.6 Apocynin

Apocynin, a natural antioxidant, stands out as a selective inhibitor of NOX, one of the main enzymes generating ROS in conditions of neuroinflammation [142]. Within this context, several *in vivo* studies have been conducted using murine models of the disease, yielding contradictory results. For instance, a study utilizing a transgenic mouse model of ALS (SOD1G93A) observed an increase in the number of neurons in the spinal cord and a 50% extension in life expectancy [143]. However, another study suggested that apocynin might offer somewhat limited benefits for SOD1G93A mice, as its administration failed to significantly prolong survival [144]. Consequently, the preclinical data remain contentious and insufficient for conducting patient trials.

##### 2.3.2.7 Iron chelation

The use of iron chelators in ALS has shown potential neuroprotection as a treatment. As demonstrated in this review, one of the pathophysiological mechanisms of ALS is the altered homeostasis of metal ions, especially iron. In this context, the following section describes the two main therapies linked to this biochemical pathway:

###### 2.3.2.7.1 M30

M30 is a novel iron chelator employed in models of neurodegenerative diseases, showcasing its capability to inhibit the enzyme monoamine oxidase (MAO), scavenge free radicals directly, induce survival signaling pathways, and restrict iron-dependent lipid peroxidation [145,146]. This positions M30 with significant potential as a neuroprotective. Within this framework, research has been undertaken to assess its effectiveness as a potential therapy for ALS.

An *in vivo* study involving SOD1G93A mice unveiled that the combination of M30 and a diet supplemented with high-calorie content substantially enhanced neurological/motor function and extended the lifespan of the mice [147]. Another study, encompassing both *in vitro* experiments with cell cultures and *in vivo* experiments with transgenic mice, yielded comparable results [145]. These investigations underscore the promise of M30 as an ALS therapy, albeit the absence of human studies at present.

###### 2.3.2.7.2 Deferiprone

Deferiprone is a commonly used iron chelator in the clinic for treating hemosiderosis. One of its notable features is its ability to traverse membranes, reduce regional iron accumulation, and redistribute captured iron to extracellular transferrin [148]. In the context of ALS, both an *in vivo* study involving transgenic mice and a pilot clinical trial with ALS patients have been undertaken. In mice, iron chelation has demonstrated the extension of life expectancy, showing a 56% increase in survival time after the onset of the disease. Furthermore, the 12-month pilot clinical trial in ALS patients revealed that deferiprone significantly lowered iron levels, oxidative stress markers, and neurofilament light chains in CSF. Additionally, during the initial 3 months of deferiprone treatment, the reduction in ALS Functional Rating Scale score was significantly less compared to the 3-month period without treatment [149]. These findings suggest that further studies should be pursued in the near future to explore iron chelation as a new neuroprotective and treatment modality for ALS patients.

## 3 Conclusion

As demonstrated in this research, there are several limitations when seeking a therapy with potential for symptomatic treatment or improving the survival of ALS patients. While most therapies tested in mouse models have shown promising results, many of these therapies evaluated in clinical trials fail to demonstrate any beneficial effects on ALS progression. In this study, several potential solutions or contributions have been proposed that could significantly aid in the development of an effective ALS therapy.

### 3.1 Pathogenesis study

Considering the recent discoveries in ALS pathogenesis, many mechanisms lack a clear explanation. It is imperative to delve deeper into these mechanisms to understand how they function and identify new potential pharmaceutical targets.

### 3.2 Better clinical characterization

Recognizing that ALS has different subtypes and that antioxidant therapies show promise in slowing disease progression, it is crucial to take an individualized approach to each patient. Rigorous disease characterization, including the assessment of pathogenic markers such as iron levels and oxidative stress markers, can help tailor therapy to each patient. Investigating the role of oxidative stress in ALS and conducting more clinical trials in various ALS subtypes is necessary to determine the most effective therapy.

### 3.3 Sporadic murine model

A significant challenge in preclinical studies is the lack of models that represent the sporadic form of ALS, which accounts for approximately 90% of cases. Most studies use SOD1 transgenic mice models, specifically the SOD1G93A mutation. Developing transgenic mouse models that mimic the sporadic presentation of the disease is essential to improve the translation of positive results from mouse models to clinical trials.

### 3.4 Multitarget antioxidant therapy

Given that oxidative stress plays a crucial role in ALS pathogenesis and involves multiple pathways for generating ROS, monotherapies may be insufficient to effectively provide neuroprotection. Several studies suggest that single-target approaches are not significantly effective. Therefore, adopting multitarget strategies where different drugs target various pathways may have a synergistic effect and slow down the disease’s progression.

A potential multitarget therapy involves the use of Riluzole, an FDA-approved drug known to have a significant impact on ALS survival rates and disease progression. Riluzole primarily functions by inhibiting glutamate presynaptic release and reducing cortical hyperexcitability [150]. By combining Riluzole with antioxidant drugs, it may be possible to address the oxidative aspects of ALS pathogenesis and potentially reduce the associated oxidative stress that contributes to disease progression (Table 1).

**Table 1:** Main antioxidant therapies that have been studied for the treatment of ALS, together with their mechanisms of action

Antioxidant therapy	Chemical name	Chemical formula	Antioxidant mechanism	References
Riluzole	6-(Trifluoromethoxy)benzothiazol-2-amine	C_8_H_5_F_3_N_2_OS	-↓ Glutamate, and inactivates voltage-dependent sodium channels	[89]
Edaravone	3-Methyl-1-phenyl-2-pyrazolin-5-one	C_10_H_10_N_2_O	-↓ linoleic acid hydroperoxide via hydroxyl radicals generated by the Fenton reaction, rather than hydroperoxide itself	[92,93]
Vitamin E	Alpha-tocopherol	C_29_H_50_O_2_	-Antioxidant effect: restricts lipid peroxidation of polyunsaturated fatty acids	[94,95]
			-Anti-inflammatory effect: inhibition of the enzyme COX-2	
NAC	N-Acetyl-L-cysteine	C_5_H_9_NO_3_S	-↑ GSH	[104,105,106]
			-Metal ion chelator and inhibition of NF-кB pathway	
CoQ10	1,4-Benzoquinone	C_59_H_90_O_4_	-Restricts lipid peroxidation and ferroptosis	[113,114]
Quercetin	2-(3,4-Dihydroxyphenyl)-3,5,7-trihydroxychromen-4-one	C_15_H_10_O_7_	-↓ Free radicals	[123,124,125]
			-↓ ER stress, apoptosis, and inflammation via SIRT1	
			-↓The formation of toxic SOD 1 fibrils	
Curcumin	(1*E*,6*E*)-1,7-bis(4-Hydroxy-3-methoxyphenyl)-1,6-heptadiene-3,5-dione	C_21_H_20_O_6_	-Activates AMPK pathway, which triggers Nrf2 signaling	[131]
			-Inhibition of NF-κB pathway	
Resveratrol	5-[(*E*)-2-(4-Hydroxyphenyl)ethenyl]benzene-1,3-diol	C_14_H_12_O_3_	-Activates SIRT1 through AMPK activation	[134,135]
Melatonin	*N*-Acetyl-5-methoxytryptamine	C_13_H_16_N_2_O_2_	-↑ GSH synthesis	[137,138]
			-Free radical scavenger and activates antioxidant enzymes SOD and GPx	
Apocynin	4-Acetyl-2-methoxyphenol	C_9_H_10_O_3_	-Selective inhibitor of NOX	[142]
M30	5-[*N*-methyl-*N*-propargylaminomethyl]-8-hydroxyquinoline	C_14_H_16_C_l2_N_2_O	-↑ mRNA expression levels of genes associated with ROS suppression	[151]
Deferiprone	1,2-Dimethyl-3-hydroxypyridin-4-one	C_7_H_9_NO_2_	-Is an iron chelator, which ↓ iron accumulation in the central motor pathways	[149]
